# Synthesis, Preliminary Bioevaluation and Computational Analysis of Caffeic Acid Analogues

**DOI:** 10.3390/ijms15058808

**Published:** 2014-05-16

**Authors:** Zhiqian Liu, Jianjun Fu, Lei Shan, Qingyan Sun, Weidong Zhang

**Affiliations:** 1 Department of Phytochemistry, School of Pharmacy, School of Pharmacy, Second Military Medical University, 325 Guo He Road, Shanghai 200433, China; E-Mails: qian12@yeah.net (Z.L.); shanleicn@126.com (L.S.); sqy_2000@163.com (Q.S.); 2 Shanghai Key Laboratory of New Drug Design, School of Pharmacy, East China University of Science and Technology, 130 Mei Long Road, Shanghai 200237, China; E-Mail: ivanliu2012@hotmail.com

**Keywords:** anti-inflammatory, caffeic acid amides, synthesis, pharmacophore, target predication

## Abstract

A series of caffeic acid amides were designed, synthesized and evaluated for anti-inflammatory activity. Most of them exhibited promising anti-inflammatory activity against nitric oxide (NO) generation in murine macrophage RAW264.7 cells. A 3D pharmacophore model was created based on the biological results for further structural optimization. Moreover, predication of the potential targets was also carried out by the PharmMapper server. These amide analogues represent a promising class of anti-inflammatory scaffold for further exploration and target identification.

## 1. Introduction

Inflammation is a hallmark of many diseases, which may lead to various diseases including sepsis, arthritis, atherosclerosis, diabetes and even cancer [1,2]. Nowadays, several steroidal anti-inflammatory drugs (SAID) and nonsteroidal anti-inflammatory drugs (NSAID) have been developed. However, they still have some unexpected side effects and the inflammation mechanism is not exactly clear. Currently, the most important targets are attracting a great deal of interest in contemporary anti-inflammatory drug design and discovery, including signal transducers and activators of transcription (STAT) [3], interleukin (IL) [4], stem cell factor (SCF) [5], macrophage migration inhibitory factor (MIF) [6], Annexin-1 [7], CC chemokine receptor (CCR) [8], Adenosine A2A receptor (A2A-R) [9], melanocortin receptor (MC-R) [10] and NF-κB signaling [11]. Very recently, our group has identified that 5-lipoxygenase was a potential target of (+)-2-(1-hydroxyl-4-oxocyclohexyl) ethyl caffeate [12].

**Figure 1.**
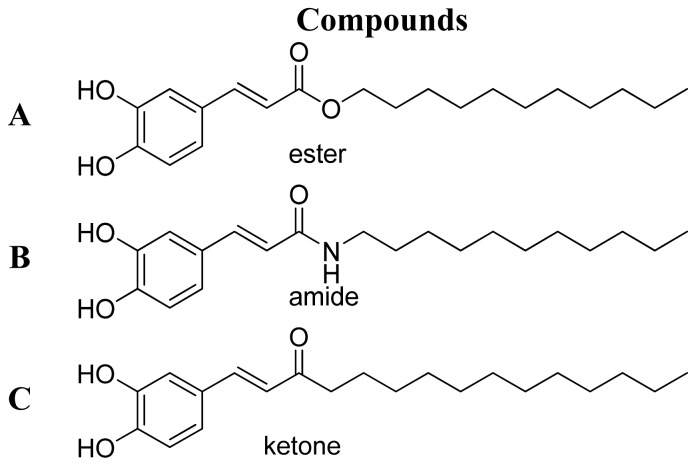
Structure of (**A**) ester; (**B**) amide; and (**C**) ketone derivatives of caffeic acid.

Caffeic acid, a common natural product from *Eucalyptus globulus* [13], *Salvinia molesta* [14], *Phellinus linteus* [15] and coffee, was reported to possess promising *in vitro* and *in vivo* anti-inflammatory properties [16]. The caffeic acid is usually found as various simple derivatives including amides, esters, sugar esters and glycosides [17]. Yuu Osanai’s group showed that caffeic acids with the ester functional group had good anti-inflammatory activity but with high cyctoxicity [18] (Figure 1). These findings prompted us to look for new caffeic acid amides with different substituent against inflammation while reducing cyctoxicity. In this report, 20 caffeic acid aimdes were rationally designed, synthesized and evaluated the inhibition of no production in murine macrophage RAW 264.7 cells. Based on the biological result, a 3D pharmacophore model was generated by using the seven active compounds with HipHop approach, which has been recognized as a time-saving and cost-effective technique for discovering new active compounds [19,20]. Furthermore, potential drug target predication was then carried out using pharmacophore-mapping approach [21]. The biological validation is ongoing now.

## 2. Results and Discussion

### 2.1. Biological Studies

**Scheme 1.**
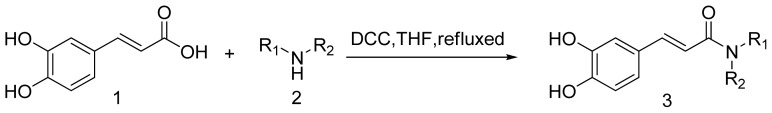
Synthetic route of the caffic acid amides.

A series of caffeic acid amides was synthesized according to general procedure [22] (Scheme 1). Firstly, R_1_ and R_2_ were first replaced with different alkyl groups (Compounds **3a**–**3f**). Unfortunately, only the *n*-Butyl derivative showed moderate nitric oxide (NO) inhibition with an IC_50_ value of 6.1 μM. The other alkyl derivatives with cyclic or di-substituted groups were completely inactive in the *in vitro* inhibition assay at 10 μM, probably due to the limited binding space (Table 1). Then, aromatic groups (Compounds **3g**–**3r**) were introduced and four compounds demonstrated good inhibitory activity. Structure−activity relationship (SAR) analysis identified that the type and position of the substituents were important for the inhibitory activity. Substituents on the 3 (Compound **3i**, IC_50_ = 7.9 μM) and 4 (Compound **3j**, IC_50_ = 5.2 μM and Compound **3k**, IC_50_ = 3.7 μM) positions of the benzene ring were favorable for the inhibition of NO production but not suitable for 3-chloro (Compound **3n**) and bromo (Compound **3o**) derivatives. Similarly, the derivatives with 2-substituents (Compounds **3l**, **3m** and **3q**) were absolutely inactive. Interestingly, the compounds with 3,5-difluorophenylo group (Compound **3h**, IC_50_ = 4.1 μM) and the 3,5-bis(trifluoromethyl)phenyl group (Compound **3g**, IC_50_ > 10 μM) were totally different. Encouraged by the above results, privileged bioactive structures with aromatic ring, such as indol (Compound **3s**) and piperonyl (Compound **3t**), were then synthesized. Both of them showed promising inhibitory activity with the IC_50_ of 6.7 and 5.0 μM, respectively, which can be taken as lead structures for further exploration. To our delight, the amides were much better than the original caffeic acid, which only had an IC_50_ value of 165 μM.

### 2.2. Pharmacophore Model

**Figure 2.**
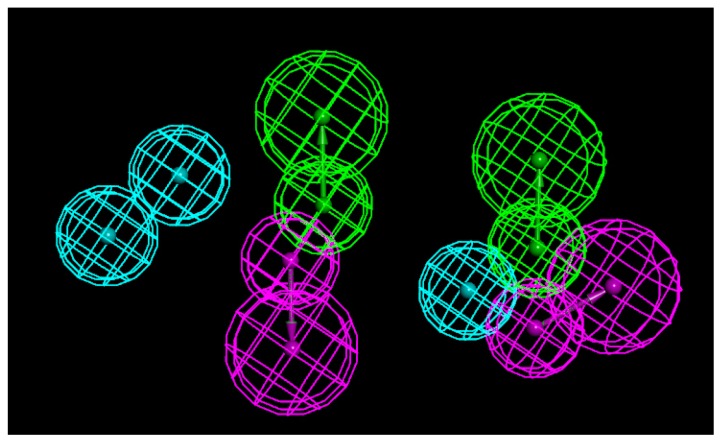
Pharmacophore model of seven active compounds. Three-dimensional spatial arrangement of the best pharmacophore hypothesis “Hypo 1”. Green color represents hydrogen bond acceptor (HBA), magenta represents hydrogen bond donor (HDB) and cyan represents hydrophobic (HY) features.

A set of the seven most potent Compounds **3a**, **3h**–**3k**, **3s** and **3t** was selected as a training set to generate the 3D pharmacophore model. The common feature pharmacophore generation run resulted in 10 pharmacophore models. All the 10 models were generated with three pharmacophoric features, along with good ranking scores ranging from 117.3 to 119.49. As all pharmacophore models didn’t have much difference in their 3D distance constraints, the best model was chosen based on the ranking score of a pharmacophore model and the fit values of the training set compounds. As a result, “Hypo 1” was selected with best ranking score of 119.49 and good fit values from the mapping of the training set compounds upon the chemical features. The pharmacophore model “Hypo 1” containing three hydrophobic (HY), two hydrogen bond acceptor (HBA) and two hydrogen bond donor (HBD) features are shown in Figure 2. The best pharmacophore model, Hypo1, was predicted using seven active compounds. It can map all seven active compounds (Figures S1–S7). The above results mimicked the 3D model of the newly synthesized active small molecules and guided further design strategy of structural optimization.

### 2.3. Target Predication and Molecular Docking

**Figure 3.**
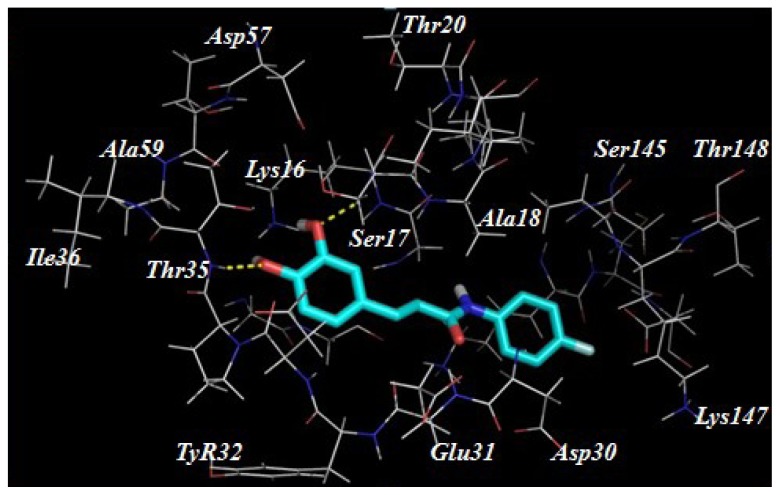
The proposed binding mode of Compound **3k** within the active site of GTPase HRas (PDB code: 5P21).

**Figure 4.**
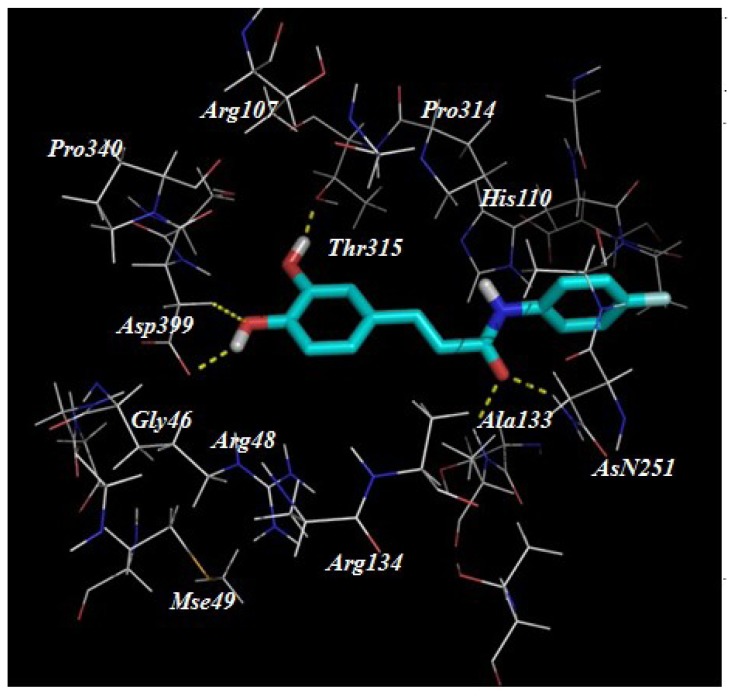
The proposed binding mode of Compound **3k** within the active site of Chorismate synthase (PDB code: 1QOX).

**Figure 5.**
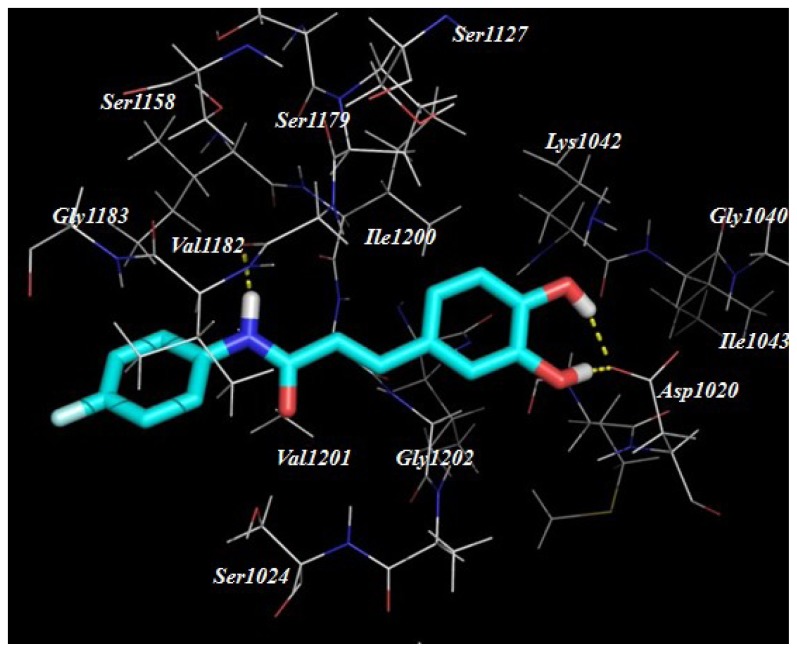
The proposed binding mode of Compound **3k** within the active site of Orotidine 5-phosphate decarboxylase (PDB code: 1LOS) and the proposed binding mode of compound **3k** within the active site of Orotidine 5-phosphate decarboxylase (PDB code: 1LOS).

Despite our synthesized compounds showed promising inhibition of NO production, the exact molecular mechanism by which exerts their effects is not yet clearly understood. Potential drug target predication was then carried out using pharmacophore-mapping approach [21]. Binding properties for Compounds **3a**, **3g**, **3h**–**3k**, **3s** and **3t** on potential targets were estimated by a reverse pharmacophore mapping server [23]. These compounds were divided into three categories: aliphatic group (Compound **3a**), aromatic group (Compounds **3h**–**3k**) and heterocyclic group (Compounds **3s** and **3t**). All the predicted targets of these three groups were ranked by the fit score. Among the top 0.3% of the predicted target candidates, there were three common targets (GTPase HRas, Chorismate synthase and Orotidine 5-phosphate decarboxylase), indicating that above compounds may target different proteins comparing with the published caffeic acid ester. Further molecular docking revealed a good interaction between the ligands and the protein active site. Compound **3k** has formed hydrogen interactions with Ser17 and Thr35 (Figure 3). In the second potential protein, it has participated in hydrogen bonds interaction with the amino acids Ala133, Asn251, Asp399 and Thr315 (Figure 4). The active site of 5-phosphate decarboxylase surrounds and binds **3k** with hydrogen bonds at Val1182 and Asp1020 (Figure 5). These three docking models supported the significance of the hydroxyl group of **3k**.

## 3. Experimental Section

### 3.1. Chemistry

All other commercial reagents and solvents were used as received without further purification. Anhydrous solvent and reagents were analytical pure and dried through routine protocols. The reactions were monitored using analytical thin layer chromatography (TLC) with Merck silica gel 60, F-254 precoated plates (0.25 mm thickness). And the TLC plates were detected under UV light. Flash column chromatography was performed with Merck silica gel 60 (Merck KGaA, Darmstadt, Germany) (200–400 mesh) or the crude product was purified by precipitation from dichloromethane with diethyl ether. ^1^H NMR and ^13^C NMR spectra were recorded on Bruker DRX 400 (Bruker Co., Bruker, Germany) at 400, 500 and 100 MHz, using TMS as an internal standard and DMSO-*d*_6_ (Sigma-Aldrich Co., St. Louis, MO, USA) as solvents. Chemical shifts (δ values) and coupling constants (*J* values) are given in ppm and Hz, respectively. ESI-MS (Agilent Technologies, Palo Alto, CA, USA) was recorded on a Waters ZQ 4000 LC-MS (Waters, Milford, MA, USA) spectrometer. The purity of the final compounds was determined using CH_3_CN/H_2_O (85:15) with 0.1% triethylamine as the mobile phase with a flow rate of 1.0 mL/min on a C_18_ column.

#### 3.1.1. General Procedure for the Preparation of Amine (**3a**–**3t**)

A solution of the caffeic acid (180 mg, 1 mmol), the dicyclohexyl carbodiimide (DCC, 206 mg, 1 mmol) and amide (1 mmol) was refluxed in THF and the progress of the reaction was monitored by TLC. The solvent was removed under vacuum. The residue was purified by flash chromatography using dichloromethane with diethyl ether (2:1–1:1) as the eluent [18].

*(E)-N-Butyl-3-(3,4-dihydroxyphenyl)acrylamide* (**3a**). Yield: 65%; ^1^H NMR (DMSO-*d*_6_, 500 MHz) δ: 9.30 (s, 1H), 9.07 (s, 1H), 7.90 (t, *J* = 5.6 Hz, 1H), 7.19 (d, *J* = 15.7 Hz, 1H), 6.91 (d, *J* = 2.0 Hz, 1H), 6.80 (dd, *J* = 8.1, 1.9 Hz, 1H), 6.71 (d, *J* = 8.1 Hz, 1H), 6.29 (d, *J* = 15.7 Hz, 1H), 3.30 (s, 2H), 3.12 (dd, *J* = 12.8, 6.8 Hz, 2H), 1.97 (s, 2H), 1.48–1.36 (m, 2H), 1.36–1.16 (m, 2H), 0.90–0.81 (m, 3H). ^13^C NMR (126 MHz, DMSO) δ: 165.6, 139.2, 126.8, 120.6, 119.0, 116.14, 114.18, 38.66, 31.75, 20.04, 14.09. ESI-MS (*m*/*z*): 236.12 [M + 1]. High performace liquid chromatograph (HPLC) purity: 97%. Anal. calcd for C_13_H_17_NO_3_: C 66.36, H 7.28, N 5.95, O 20.40, found: C 66.27, H 7.18, N 5.90.

*(E)-N-(Cyclopropylmethyl)-3-(3,4-dihydroxyphenyl)acrylamide* (**3b**). Yield: 55%; ^1^H NMR (DMSO-*d*_6_, 400 MHz) δ: 9.36 (s, 1H), 9.13 (s, 1H), 8.05–8.08 (m, 1H), 7.22 (d, *J* = 16 Hz, 1H), 6.94 (s, 1H), 6.83 (d, *J* = 8.0 Hz, 1H), 6.74 (d, *J* = 8.4 Hz, 1H), 6.35 (d, *J* = 16.0 Hz, 1H), 3.04 (t, *J* = 6.0 Hz, 2H), 0.40–0.44 (m, 2H), 0.16–0.19 (m, 2H). ESI-MS (*m*/*z*): 234.11 [M + 1]. HPLC purity: 96.5%.

*(E)-3-(3,4-Dihydroxyphenyl)-1-(piperidin-1-yl)prop-2-en-1-one* (**3c**). Yield: 35%; ^1^H NMR (DMSO-*d*_6_, 400 MHz) δ: 9.42 (s, 1H), 8.97 (s, 1H), 7.3 (d, *J* = 15.2 Hz, 1H), 7.08 (s, 1H), 6.89–6.98 (m, 2H), 6.73 (d, *J* = 15.2 Hz, 1H), 3.51–3.59 (m, br, 4H), 1.48–1.60 (m, 6H). ESI-MS (*m*/*z*): 248.02 [M + 1]. HPLC purity: 98%.

*(E)-3-(3,4-Dihydroxyphenyl)-1-(pyrrolidin-1-yl)prop-2-en-1-one* (**3d**). Yield: 45%; ^1^H NMR (DMSO-*d*_6_, 400 MHz) δ: 9.47 (s, 1H), 9.10 (s, 1H), 7.29 (d, *J* = 15.2 Hz, 1H), 7.05 (s, 1H), 6.96 (d, *J* = 8.0 Hz, 1H), 6.74 (d, *J* = 7.6 Hz, 1H), 6.64 (d, *J* = 15.2 Hz, 1H), 3.59 (t, *J* = 6.0 Hz, 2H), 3.67 (t, *J* = 6.0 Hz, 2H), 1.90 (m, 2H), 1.79 (m, 2H). ESI-MS (*m*/*z*): 234.1 [M + 1]. HPLC purity: 97%.

*(E)-1-(Aziridin-1-yl)-3-(3,4-dihydroxyphenyl)prop-2-en-1-one* (**3e**). Yield: 69%; ^1^H NMR (DMSO-*d*_6_, 400 MHz) δ: 9.37 (s, 1H), 9.14 (s, 1H), 7.22 (d, *J* = 15.6 Hz, 1H), 6.92 (s, 1H), 6.82 (d, *J* = 8.0 Hz, 1H), 6.73 (d, *J* = 8.0 Hz, 1H), 6.23 (d, *J* = 15.6 Hz, 1H), 0.64–0.67 (m, 2H), 0.42–0.43 (m, 2H). ESI-MS (*m*/*z*): 206.07 [M + 1]. HPLC purity: 97.2%.

*(E)-N,N-Dibutyl-3-(3,4-dihydroxyphenyl)acrylamide* (**3f**). Yield: 57%; ^1^H NMR (DMSO-*d*_6_, 400 MHz) δ: 9.41 (s, 1H), 9.04 (s, 1H), 7.30 (d, *J* = 15.2 Hz, 1H), 7.04 (s, 1H), 6.94 (d, *J* = 8.0 Hz, 1H), 6.73–6.77 (m, 2H), 3.41 (t, *J* = 7.2 Hz, 2H), 3.30(t, *J* = 7.2 Hz, 2H), 1.0–1.5 (m, 8H), 0.87–0.94 (m, 6H). ESI-MS (*m*/*z*): 292.02 [M + 1]. HPLC purity: 98%.

*(E)-N-(3,5-Bis(trifluoromethyl)phenyl)-3-(3,4-dihydroxyphenyl)acrylamide* (**3g**). Yield: 90%; 1H NMR (DMSO-*d*_6_, 400 MHz) δ: 10.46 (s, 1H), 9.50 (s, 1H), 9.24 (s, 1H), 7.39–7.48 (m, 3H), 7.02 (s, 1H), 6.88–6.94 (m, 2H), 6.78 (d, *J* = 8.0 Hz, 1H), 6.47 (d, *J* = 15.2 Hz, 1H). ESI-MS (*m*/*z*): 392.06 [M + 1]. HPLC purity: 96%.

*(E)-N-(3,5-Difluorophenyl)-3-(3,4-dihydroxyphenyl)acrylamide* (**3h**). Yield: 71%; ^1^H NMR (DMSO-*d*_6_, 500 MHz) δ: 7.46–7.22 (m, 3H), 7.00 (d, *J* = 1.7 Hz, 1H), 6.95–6.80 (m, 2H), 6.76 (d, *J* = 8.1 Hz, 1H), 6.45 (d, *J* = 15.6 Hz, 1H). ^13^C NMR (126 MHz, DMSO) δ: 164.9, 163.7, 161.9, 148.5, 146.0, 142.4, 126.2, 121.5, 116.2, 114.4, 102.2. ESI-MS (*m*/*z*): 292.07 [M + 1]. HPLC purity: 97.2%. Anal. calcd for C_15_H_11_F_2_NO_3_: C 61.86, H 3.81, F 13.05, N 4.81, O 16.48, found: C 61.76, H 3.80, F 13.00, N 4.69, O 16.27.

*(E)-3-(3,4-Dihydroxyphenyl)-N-(3-(trifluoromethyl)phenyl)acrylamide* (**3i**). Yield: 59%; ^1^H NMR (DMSO-*d*_6_, 500 MHz) δ: 8.18 (s, 1H), 7.83 (d, *J* = 8.4 Hz, 1H), 7.53 (dd, *J* = 14.6, 6.6 Hz, 1H), 7.43 (d, *J* = 15.6 Hz, 1H), 7.37 (d, *J* = 7.7 Hz, 1H), 7.00 (d, *J* = 1.9 Hz, 1H), 6.91 (d, *J* = 8.2, 2.0 Hz, 1H), 6.76 (d, *J* = 8.1 Hz, 1H), 6.50 (d, *J* = 15.6 Hz, 1H). ^13^C NMR (126 MHz, DMSO) δ: 164.91, 148.38, 146.06, 142.00, 140.66, 130.40, 126.38, 122.99, 121.45, 119.73, 118.14–117.25, 116.24, 115.50, 114.43. ESI-MS (*m*/*z*): 324.08 [M + 1]. HPLC purity: 98%. Anal. calcd for C_16_H_12_F_3_NO_3_:C 59.45, H 3.74, F 17.63, N 4.33, O 14.85, found: C59.35, H 3.50, F 17.53, N 4.31, O 14.65.

*(E)-3-(3,4-Dihydroxyphenyl)-N-(4-methoxyphenyl)acrylamide* (**3j**). Yield: 78%; ^1^H NMR (DMSO-*d*_6_, 500 MHz) δ: 9.90 (s, 1H), 7.58 (d, *J* = 9.0 Hz, 2H), 7.35 (d, *J* = 15.6 Hz, 1H), 6.98 (d, *J* = 1.8 Hz, 1H), 6.90–6.82 (m, 3H), 6.75 (d, *J* = 8.1 Hz, 1H), 6.49 (d, *J* = 15.6 Hz, 1H), 3.71 (s, 3H). ^13^C NMR (DMSO-d_6_, 126 MHz,) δ: 163.9, 155.5, 147.9, 146.0, 140.6, 133.1, 126.7, 121.0, 118.99 (s, 3H), 116.2, 114.3, 55.5. ESI-MS (*m*/*z*): 286.1 [M + 1]. HPLC purity: 96.6%. Anal. calcd for C_15_H_16_NO_4_: C 67.36, H 5.34, N 4.91, O 22.43, found: C 67.20, H 5.22, N 4.90. O 22.25.

*(E)-3-(3,4-Dihydroxyphenyl)-N-(4-fluorophenyl)acrylamide* (**3k**). Yield: 61%; ^1^H NMR (DMSO-*d*_6_, 500 MHz) δ: 10.09 (s, 1H), 9.33 (br, 2H), 7.68 (d, *J* = 14.1 Hz, 2H), 7.38 (d, *J* = 15.6 Hz, 1H), 7.13 (d, *J* = 15.6 Hz, 2H), 6.98 (s, 1H), 6.88 (dt, *J* = 15.2, 7.6 Hz, 1H), 6.75 (d, *J* = 8.1 Hz, 1H), 6.48 (d, *J* = 6.0 Hz, 1H). ^13^C NMR (DMSO-*d*_6_, 126 MHz) δ: 164.3, 159.2, 157.3, 146.0, 141.2, 121.2, 118.6, 116.2, 115.83, 115.7, 114.3. ESI-MS (*m*/*z*): 274.08 [M + 1]. HPLC purity: 97.4%. Anal. calcd for C_15_H_12_FNO_3_:C 65.93, H 4.43, F 6.95, N 5.13, O 17.57, found: C 65.65, H 4.35, F 6.72, N 5.05, O 17.37.

*(E)-3-(3,4-Dihydroxyphenyl)-N-(2-(hydroxymethyl)phenyl)acrylamide* (**3l**). Yield: 63%; ^1^H NMR (DMSO-*d*_6_, 400 MHz) δ: 9.48 (s, 1H), 9.46 (s, 1H), 9.17 (s, 1H), 7.76 (d, *J* = 4.0 Hz, 1H), 7.36–7.43 (m, 2H), 7.25 (t, *J* = 8.0 Hz, 1H), 7.15 (t, *J* = 8.0 Hz, 1H), 7.03 (s, 1H), 6.92 (d, *J* = 8.8 Hz, 1H), 6.77 (d, *J* = 8.4 Hz, 1H), 6.60 (d, *J* = 15.2 Hz, 1H), 4.53 (s, 2H). ESI-MS (*m*/*z*): 286.1 [M + 1]. HPLC purity: 97.6%.

*(E)-N-(2-Acetylphenyl)-3-(3,4-dihydroxyphenyl)acrylamide* (**3m**). Yield: 53%; ^1^H NMR (DMSO-*d*_6_, 400 MHz) δ: 11.39 (s, 1H), 9.55 (s, 1H), 9.16 (s, 1H), 8.42 (d, *J* = 8.0 Hz, 1H), 8.00 (d, *J* = 8.0 Hz, 1H), 7.62 (t, *J* = 7.6 Hz, 2H), 7.44 (d, *J* = 15.2 Hz, 1H), 7.22 (t, *J* = 7.6 Hz, 1H), 7.09 (s, 1H), 7.01 (d, *J* = 8.0 Hz, 1H), 6.78 (d, 2H, *J* = 8.0 Hz), 6.53 (d, *J* = 15.2 Hz, 1H), 2.64 (s, 3H). ESI-MS (*m*/*z*): 298.1 [M + 1]. HPLC purity: 97.8%.

*(E)-N-(3-Chlorophenyl)-3-(3,4-dihydroxyphenyl)acrylamide* (**3n**). Yield: 72%; ^1^H NMR (DMSO-*d*_6_, 400 MHz) δ: 10.27 (s, 1H), 9.51 (s, 1H), 9.23 (s, 1H), 7.93 (s, 1H), 7.51 (d, *J* = 8.0 Hz, 1H), 7.42 (d, *J* = 15.2 Hz, 1H), 7.36 (t, *J* = 8.0 Hz, 1H), 7.10 (d, *J* = 8.0 Hz, 1H), 7.01 (s, 1H), 6.92 (d, *J* = 8.0 Hz, 1H), 6.78 (d, *J* = 8.0 Hz, 1H), 6.50 (d, *J* = 15.2 Hz, 1H). ESI-MS (*m*/*z*): 290.02 [M + 1]. HPLC purity: 97.9%.

*(E)-N-(3-Bromophenyl)-3-(3,4-dihydroxyphenyl)acrylamide* (**3o**). Yield: 67%; ^1^H NMR (DMSO-*d*_6_, 400 MHz) δ: 10.24 (s, 1H), 9.51 (s, 1H), 9.22 (s, 1H), 8.06 (s, 1H), 7.55 (d, *J* = 8.0 Hz, 1H), 7.42 (d, *J* = 15.2 Hz, 1H), 7.22–7.30 (m, 2H), 7.01 (s, 1H), 6.92 (d, *J* = 8.0 Hz, 1H), 6.77 (d, *J* = 8.0 Hz, 1H), 6.50 (d, *J* = 15.2 Hz, 1H). ESI-MS (*m*/*z*): 334.16 [M + 1]. HPLC purity: 97.6%.

*(E)-3-(3,4-Dihydroxyphenyl)-N-p-tolylacrylamide* (**3p**). Yield: 76%; ^1^H NMR (DMSO-*d*_6_, 400 MHz) δ: 9.98 (s, 1H), 9.44 (s, 1H), 9.20 (s, 1H), 7.57 (d, *J* = 6.8 Hz, 2H), 7.38 (d, *J* = 15.2 Hz, 1H), 7.20 (d, *J* = 6.8 Hz, 2H), 7.00 (s, 1H), 6.90 (d, *J* = 8.0 Hz, 1H), 6.77 (d, *J* = 8.0 Hz, 1H), 6.52 (d, *J* = 15.2 Hz, 1H), 2.26 (s, 3H). ESI-MS (*m*/*z*): 270.11 [M + 1]. HPLC purity: 97.3%.

*(E)-3-(3,4-Dihydroxyphenyl)-N-o-tolylacrylamide* (**3q**). Yield: 68%; ^1^H NMR (DMSO-d_6_, 400 MHz) δ: 9.45 (s, 1H), 9.32 (s, 1H), 9.17 (s, 1H), 7.58 (d, *J* = 8.0 Hz, 1H), 7.38 (d, *J* = 15.2 Hz, 1H), 7.16–7.23 (m, 2H), 7.01–7.08 (m, 2H), 6.91 (d, *J* = 8.0 Hz, 1H), 6.77 (d, *J* = 8.0 Hz, 1H), 6.68 (d, *J* = 15.2 Hz, 1H), 2.24 (s, 3H). ESI-MS (*m*/*z*): 270.1 [M + 1]. HPLC purity: 96%.

*(E)-N-Benzyl-3-(3,4-dihydroxyphenyl)-N-methylacrylamide* (**3r**). Yield: 64%; ^1^H NMR (DMSO-*d*_6_, 400 MHz) δ: 9.41 (s, 1H), 9.00 (s, 1H), 7.21–7.40 (m, 6H), 6.89–7.01 (m, 3H), 6.71–6.76 (m, 1H), 4.69 (s, 2H), 2.98 (s, 3H). ESI-MS (*m*/*z*): 284.12 [M + 1]. HPLC purity: 97%.

*(E)-N-(2-(1H-Indol-3-yl)ethyl)-3-(3,4-dihydroxyphenyl)acrylamide* (**3s**). Yield: 91%; ^1^H NMR (DMSO-*d*_6_, 500 MHz) *δ*: 10.78 (s, 1H), 9.20 (d, *J* = 101.6 Hz, 2H), 8.07 (t, *J* = 5.7 Hz, 1H), 7.54 (d, *J* = 7.9 Hz, 1H), 7.32 (d, *J* = 8.1 Hz, 1H), 7.23 (d, *J* = 15.6 Hz, 1H), 7.14 (s, 1H), 7.04 (dd, *J* = 11.1, 4.0 Hz, 1H), 7.00–6.88 (m, 2H), 6.81 (dd, *J* = 8.2, 1.9 Hz, 1H), 6.73 (d, *J* = 8.1 Hz, 1H), 6.32 (d, *J* = 15.7 Hz, 1H), 3.44 (d, *J* = 13.4, 7.1 Hz, 2H), 2.86 (t, *J* = 7.4 Hz, 2H). ^13^C NMR (DMSO-*d*_6_, 126 MHz) δ: 165.7, 147.6, 145.9, 139.3, 136.6, 127.6, 126.8, 123.0, 121.3, 120.7, 119.1, 118.6, 116.1, 114.2, 112.2, 111.7, 31.0, 25.7. ESI-MS (*m*/*z*): 323.13 [M + 1]. HPLC purity: 98%. Anal. calcd for C_20_H_20_N_2_O_3_: C 71.41, H 5.99, N 8.33, O 14.24, found: C 71.26, H 5.55, N 8.12, O 14.17.

*(E)-N-(2-(Benzo[d][1,3]dioxol-5-yl)ethyl)-3-(3,4-dihydroxyphenyl)acrylamide* (**3t**). Yield: 52%; ^1^H NMR (DMSO-*d*_6_, 500 MHz) δ: 9.37 (s, 1H), 9.14 (s, 1H), 8.03 (t, *J* = 5.5 Hz, 1H), 7.22 (d, *J* = 15.7 Hz, 1H), 6.93 (s, 1H), 6.82 (d, *J* = 7.9 Hz, 3H), 6.73 (d, *J* = 8.1 Hz, 1H), 6.67 (d, *J* = 8.0 Hz, 1H), 6.31 (d, *J* = 15.7 Hz, 1H), 5.96 (s, 2H), 3.37–3.29 (m, 4H), 2.69 (dd, *J* = 18.5, 11.2 Hz, 2H).^13^C NMR (DMSO-*d*_6_, 126 MHz) δ: 165.7, 161.3, 147.5, 145.9, 139.3, 133.5, 126.8, 121.9, 120.7, 118.9, 116.1, 114.2, 109.4, 108.5, 101.0, 35.2, 35.0. ESI-MS (*m*/*z*): 228.3 [M + 1]. HPLC purity: 97.7%. Anal. calcd for C_18_H_17_NO_5_: C 66.05, H 5.23, N 4.28, O 24.44, found: C 65.95, H 5.13, N 4.15, O 24.26.

### 3.2. Biology

#### 3.2.1. Cell Culture

RAW 264.7 murine macrophages were obtained from the Shanghai Institute of Cell Biology, Chinese Academy of Sciences (Shanghai, China) and maintained in DMEM recommended by the suppliers, supplemented with 10% fetal bovine serum (Gibco, Paisley, UK), penicillin (100 U/mL) and streptomycin (100 mg/mL) in a humidified 5% CO_2_ atmosphere at 37 °C.

#### 3.2.2. Measurement of Nitric Oxide

The amount of NO was assessed by determining the nitrite concentration with Griess reagent. Briefly, in the experiment to assess NO in culture supernatants, RAW 264.7 macrophages were seeded into 48-well plates (2 × 10^6^ cells per mL) for 18 h. Then, the cells were pretreated each sample, aminoguanidine or vehicle solution for 20 min, then stimulated with LPS (1 μg/mL) for 18 h. Samples of supernatants (100 mL) were incubated with 50 mL 1% sulfanilamide, then 50 mL of 0.1% naphthylethylenediamine in 2.5% phosphoric acid solution. The absorbance at 570 nm was read and referred to a standard curve of sodium nitrite solution to determine the nitrite concentration. In the other experiment to determine the NO concentration of exudates from rat air pouches, the exudates (50 mL) were incubated with nitrate reductase solution (200 mL; Jiancheng Bioengineering Institute, Nanjing, China) at 37 °C for 1 h. Nitrate was converted into nitrite. After centrifugation, the nitrite concentration in the cell-free supernatants was assessed with Griess reagent as described above [24].

### 3.3. Computational Protocols

#### 3.3.1. Pharmacophore Generation

All the studies were carried out using Discovery Studio (DS) 2.5 unless it is mentioned. Seven most active compounds as shown in Figure 2 were selected as a training set to generate qualitative pharmacophore models to be used in future database screening to identify new scaffolds for drug discovery. The 2D chemical structures of the training set compounds were built using ChemSketch program version 12, and subsequently converted into 3D structures using DS. All compounds in the training set were given a Principal value of 2 and a Maximum Omitted Feature value of 0 to make sure that all the features of these compounds are considered during pharmacophore generation. Diverse conformational models for every training set compound were generated to cover the flexibility of their chemical nature using polling algorithm. All the compounds were energetically minimized using CHARMM force field implemented in DS. Diverse Conformation Generation protocol with BEST flexible search option implemented in DS was employed with the default value of generating maximum of 250 conformers within the energy range of 20 kcal/mol, with respect to the global minimum. Feature mapping protocol was employed prior to the original pharmacophore generation calculation to identify the chemical features present in the training set compounds. The chemical features such as hydrogen bond acceptor (HBA), hydrogen bond donor (HBD) and hydrophobic (HY) features were used during pharmacophore generation. These chemical features were selected based on the feature mapping results and the possible interaction points. All the other parameters were maintained at their default settings. The seven compounds in the training set along with the generated conformational models were used in pharmacophore model generation. Common feature pharmacophore models, generally, are developed by comparing a set of conformational models and a number of 3D configurations of chemical features shared among the training set compounds. Common Feature Pharmacophore Model Generation protocol implemented in DS was used to generate pharmacophore models. Minimum interfeature distance was 0.5. The other parameters were default.

#### 3.3.2. Molecular Docking Study

All the molecular docking studies were performed by GOLD 5.1 program with ChemPLP function score (Cambridge Crystallographic Data Center, London, UK). X-ray crystal structures (PDB ID: 1LOS, PDB ID: 1QXO, PDB ID: 5P21) were used to define the binding site for molecular docking studies. The radius of 12 Å around the active compound was defined to form the active site of the protein.

## 4. Conclusions

In summary, we rationally designed a series of caffeic acid amide analogues. The preliminary biological evaluations revealed that this class of compounds possessed moderate to good anti-inflammatory activity. A 3D pharmacophore model was then generated based on the biological activity and the better understanding of this feature could provide meaningful insights for further optimization. Potential targets were also predicted by the PharmMapper server. A further study of the structural modification and biological target validation are in process in our laboratory and will be reported elsewhere.

## Supplementary Information



## Figures and Tables

**Table 1.** Synthesis of caffeic acid amide (**3a**–**3t**) and inhibitory effect of caffeic acid amides on Lipopolysaccharide (LPS) induced nitrite production.

Compounds	R_1_	R_2_	Nitric Oxide Inhibition/IC_50_ (μM)
**f**	*n*-butyl	H	6.1
**3b**	cyclopropylmethanyl	H	>10
**3c**	–CH_2_)_5_–	–(CH_2_)_5_–	>10
**3d**	–(CH_2_)_4_–	–(CH_2_)_4_–	>10
**3e**	–(CH_2_)_2_–	–(CH_2_)_2_–	>10
**3f**	*n*-butyl	*n*-butyl	>10
**3g**	3,5-bis(trifluoromethyl)phenyl	H	>10
**3h**	3,5-difluorophenyl	H	4.1
**3i**	3-(trifluoromethyl)phenyl	H	7.9
**3j**	4-methoxyphenyl	H	5.2
**3k**	4-fluorophenyl	H	3.7
**3l**	2-(hydroxymethyl)phenyl	H	>10
**3m**	2-acetylphenyl	H	>10
**3n**	3-chlorophenyl	H	>10
**3o**	3-bromophenyl	H	>10
**3p**	4-methanylphenyl	H	>10
**3q**	2-methanylphenyl	H	>10
**3r**	phenylmethanyl	H	>10
**3s**	2-(1H-indol-3-yl)ethyl	H	6.7
**3t**	2-(benzo[d][1,3]dioxol-5-yl)ethyl	H	5.0

caffeic acid	-	-	165 a

a data from the reference [2].
