# Supplementary material for: Synthesis, Preliminary Bioevaluation and Computational Analysis of Caffeic Acid Analogues
Source: Int J Mol Sci. 2014 May 16;15(5):8808–20. doi: 10.3390/ijms15058808 (PMC4057760; doi:10.3390/ijms15058808)
Supplement: Supplementary file 1 [file ijms-15-08808-s001.pdf]

Supplementary Information

Figure S1. Mapping of active compound **3a** in the pharmacophore.

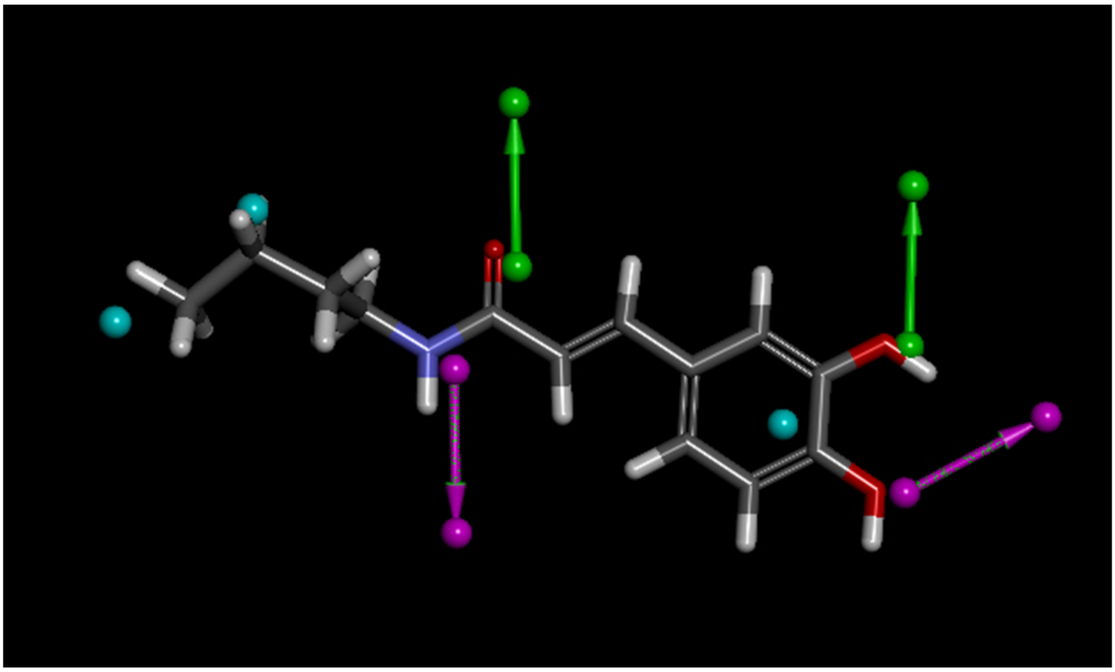

Figure S2. Mapping of active compound **3h** in the pharmacophore.

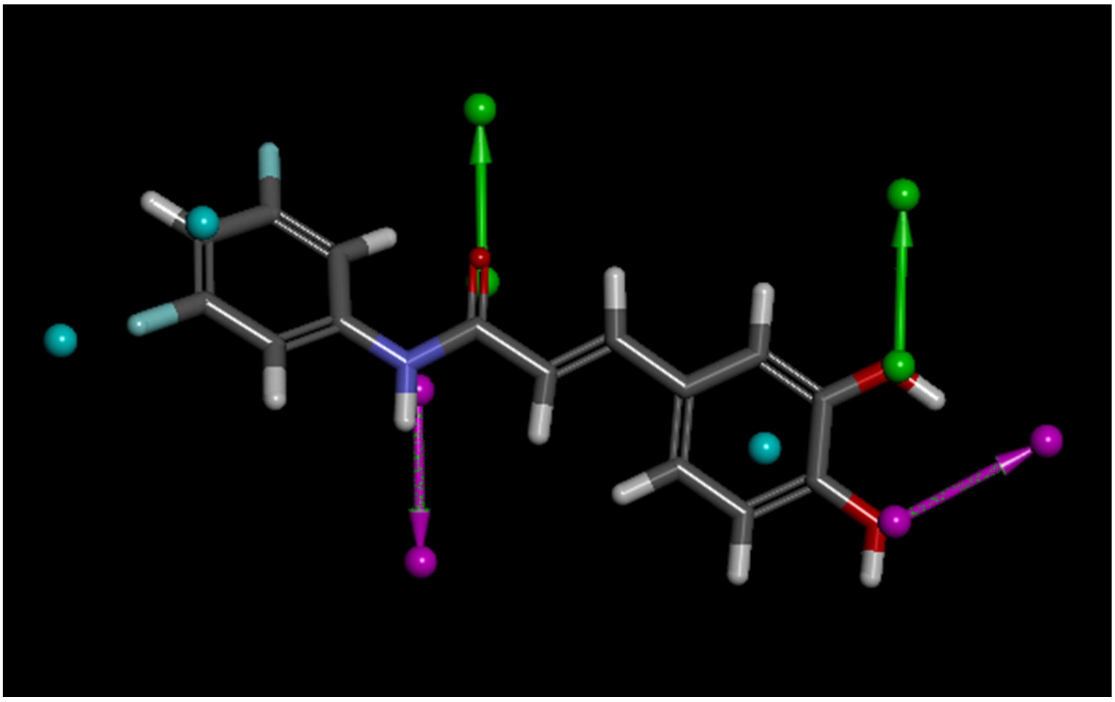

Figure S3. Mapping of active compound **3i** in the pharmacophore.

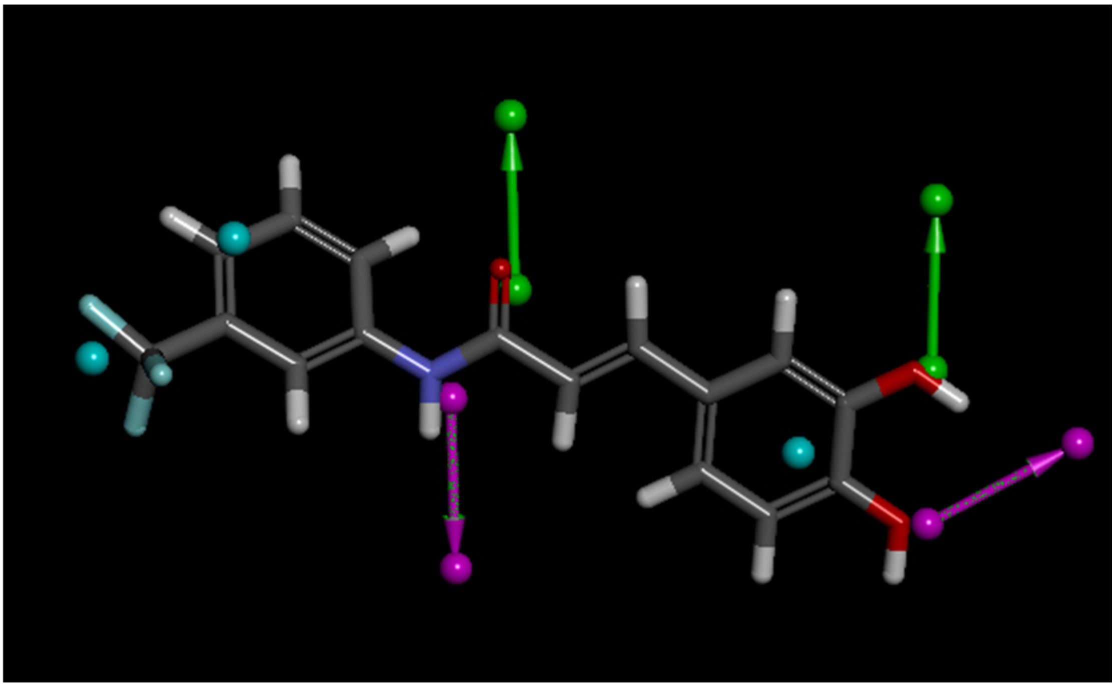

Figure S4. Mapping of active compound **3j** in the pharmacophore.

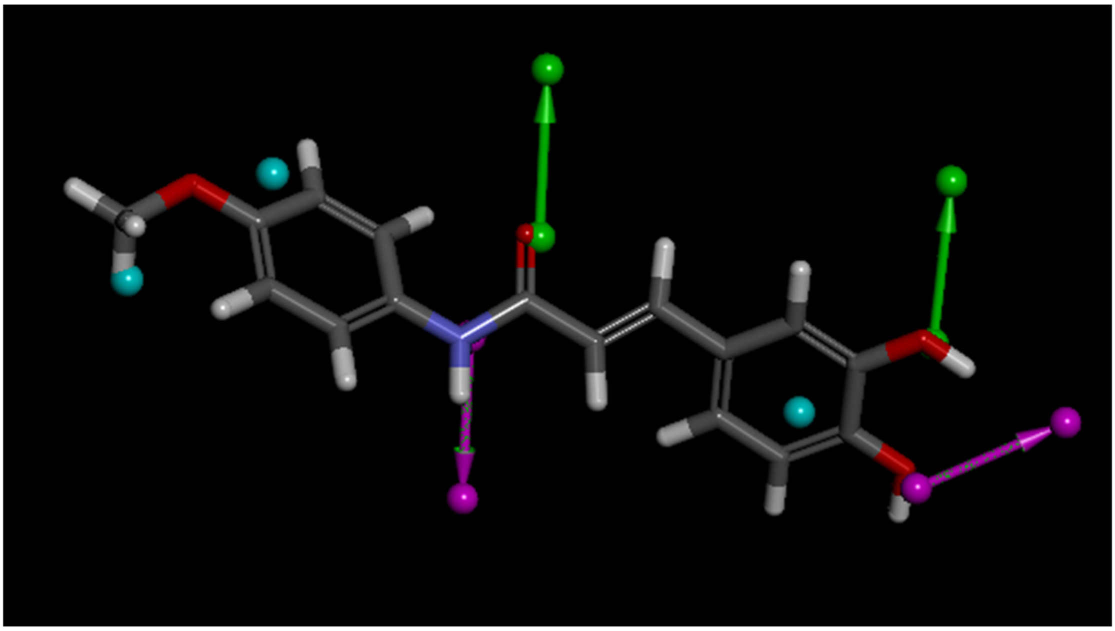

Figure S5. Mapping of active compound **3k** in the pharmacophore.

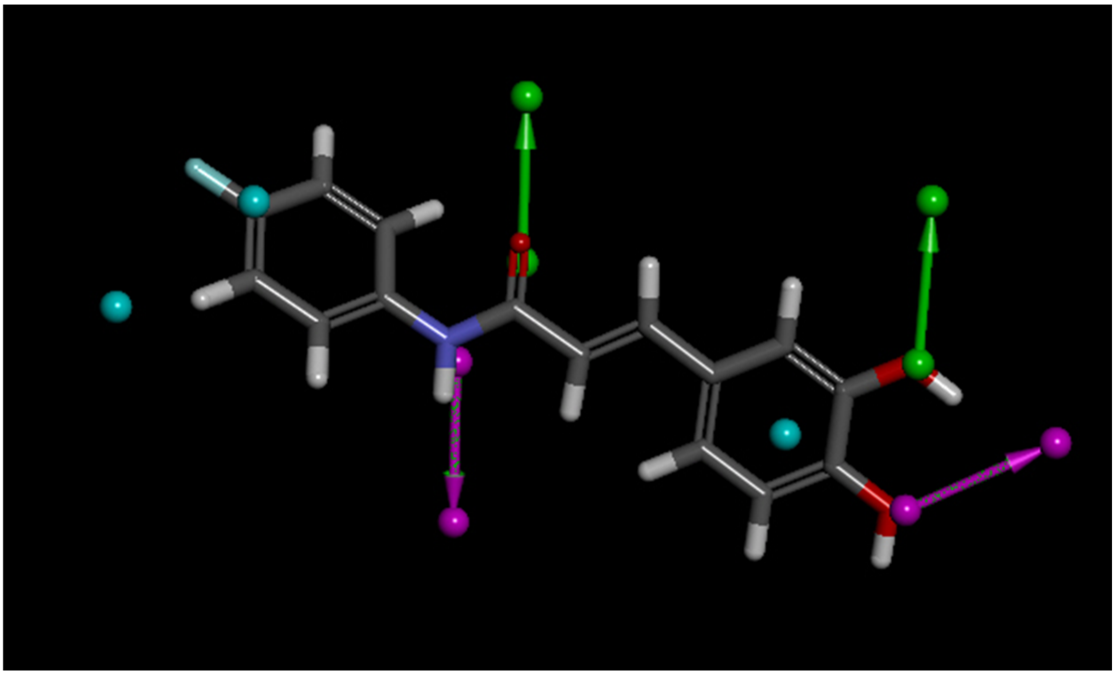

Figure S6. Mapping of active compound **3s** in the pharmacophore.

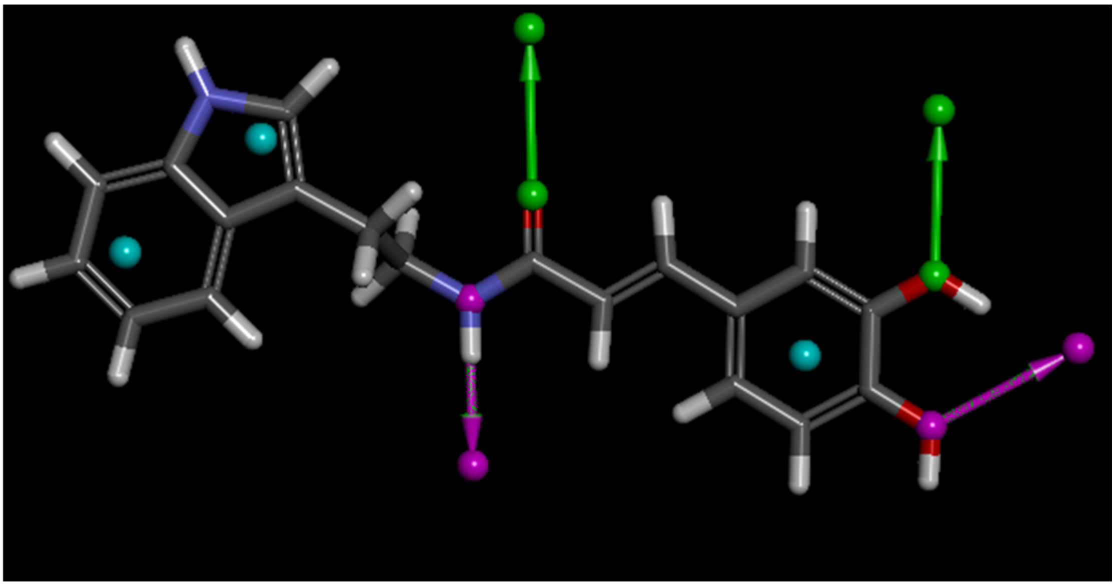

Figure S7. Mapping of active compound **3t** in the pharmacophore.

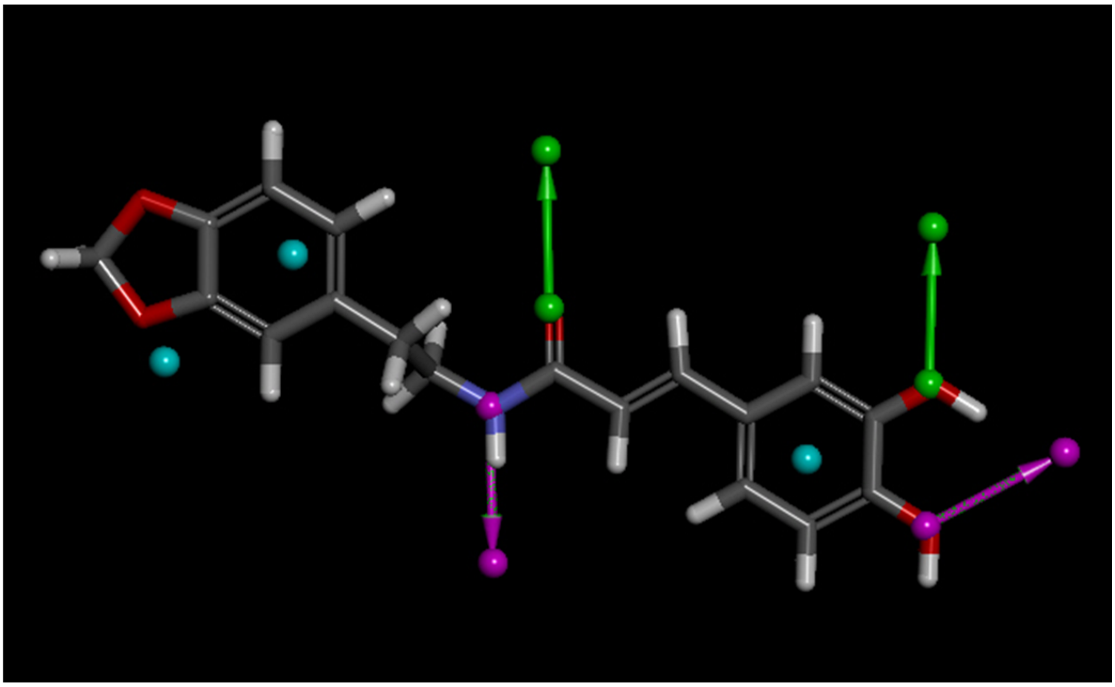

© 2014 by the authors; licensee MDPI, Basel, Switzerland. This article is an open access article distributed under the terms and conditions of the Creative Commons Attribution license (http://creativecommons.org/licenses/by/3.0/).
